# The Refractive Index of Human Milk Serum: Natural Variations and Dependency on Macronutrient Concentrations

**DOI:** 10.3390/foods13244124

**Published:** 2024-12-20

**Authors:** Johanna R. de Wolf, Kawthar Ali, Chris G. Legtenberg, Wietske Verveld, Nienke Bosschaart

**Affiliations:** Department of Biomedical Photonic Imaging, Faculty of Science and Technology, University of Twente, P.O. Box 217, 7500 AE Enschede, The Netherlandsn.bosschaart@utwente.nl (N.B.)

**Keywords:** human milk, breastmilk, refractive index, refractometry, physical property, milk serum, milk whey, milk soluble fraction, foremilk, hindmilk

## Abstract

The refractive index (RI) of human milk serum (also known as whey, milk soluble fraction or milk plasma) depends on the individual molecular species dissolved in the serum and their concentrations. Although the human milk serum RI is known to influence milk analysis methods based on light scattering, the RI dependency on human milk serum composition is currently unknown. Therefore, we systematically evaluate how the RI depends on natural variations in macronutrient concentrations in the soluble fraction. We measure RI variations in serum simulating samples with controlled macronutrient concentrations, as well as skimmed and whole fore-, bulk, and hindmilk from 19 donors. For both types of samples, we relate the measured RI to the macronutrient composition. From the serum simulating samples, we observe that the RI depends more on variations in whey protein, than carbohydrate concentrations, while minerals have negligible influence. For all donated samples, the average RI was 1.3470 (range 1.3466–1.3474). Per donor, no significant differences were observed in RI between fore-, bulk, and hindmilk. We conclude that protein and solids-not-fat (i.e., the total contribution of carbohydrates, proteins and minerals present in milk) concentrations are most predictive for human milk serum RI.

## 1. Introduction

Research into human milk composition is relevant for the fundamental understanding of milk synthesis and secretion by the mammary gland [1], milk uptake/digestion by the infant [2], infant development, and the health benefits of human milk [2]. From a practical perspective, the assessment of milk composition is desirable in various situations, ranging from milk fortification for premature infants [3,4] to mapping the changes in the macronutrient content of human milk and its relation to lactation characteristics (i.e., lactation period, number of feedings during the day, time during the day) [5].

Commonly used methods to determine the macronutrient composition in human milk are labour-intensive and destructive, hindering the possibility for inline analysis and reuse of milk for infant feeding after analysis. Chemical methods include Mojonnier ether extraction [6,7] or the Röse–Gottlieb method [8,9] for fat concentration measurements, high pressure liquid chromatography (HPLC) for lactose concentration measurements [7,8], and the Kjeldahl method [7,8,9] or bicinchoninic acid (BCA) protein assay kit [9] for protein concentration measurements. But also faster, light absorption-based infrared human milk analysers require sample sonification and homogenization prior to analysis [6,8,9]. Optical methods based on light scattering can potentially offer a rapid, non-destructive and inexpensive alternative for the analysis of human milk composition [10,11,12,13,14]. These methods are particularly sensitive to fat content, as milk fat globules are the most dominant light scattering particles in human milk. Examples of these optical methods are diffuse reflectance spectroscopy [11,15], optical coherence tomography [11,16], collimated transmission and integrating sphere measurements [13,14], particle size measurements by laser diffraction [17,18,19], and laser polarimetric scatterometry [20,21]. However, these methods inherently require knowledge about the refractive index of human milk serum, which depends on the individual molecular species dissolved in the serum and their concentrations [22,23].

Human milk serum is defined as the dispersion medium or soluble fraction of milk from which all light-scattering particles, i.e., milk fat globules, casein micelles, cells, and extracellular vesicles, have been removed [24]. Milk serum contains dissolved carbohydrates, whey proteins, vitamins, and minerals. Other frequently used terms for milk serum are milk plasma, milk soluble fraction, or whey. Whereas human milk whey is most often defined as the soluble fraction after removal of casein particles [24], bovine milk whey is commonly defined as the liquid that remains after milk is curdled and strained [25,26]. To avoid confusion, we therefore use the term milk serum in this study for the soluble fraction of milk absent of casein.

So far, the refractive index of whole human milk from 15 donors was reported and compared to the refractive index of formula milk and bovine milk [23]. However, the refractive index of human milk serum and its dependency on serum composition is still unknown, as well as potential refractive index changes during a single breastfeed. Therefore, our goal is to measure the variation in the refractive index of human milk serum during a single breastfeed and to identify the relation between the refractive index and the individual dissolved macronutrient concentrations.

## 2. Materials and Methods

### 2.1. Study Design

To systematically analyse the influence of the individual milk serum constituents on the refractive index, we measured the refractive index of serum simulating samples with known concentrations of the most abundant carbohydrates, whey proteins, and minerals in human milk. Next to that, we measured the range in refractive indices and macronutrient concentrations in donated milk samples from 19 donors with a lactation period between 1 and 9 months. Three samples were obtained per donor (n = 57 samples), which included milk released at the start of the breastfeed (foremilk), at the end of the breastfeed (hindmilk), and a fraction of the total extracted milk volume (bulk milk).

### 2.2. Serum Simulating Samples

A series of human milk serum simulating samples was created with biologically relevant variations in the concentrations of the main dissolved constituents. Table 1 lists a detailed overview of these most abundant dissolved constituents per macronutrient (i.e., carbohydrates, whey proteins, and minerals) and their range of concentrations in mature human milk (>30 days postpartum), as found in the literature.

In total, we prepared 80 serum simulating samples with different concentrations of macronutrients in the biologically relevant range. We approximate the milk serum macronutrient concentrations by including only the main constituents of milk. To create the serum simulating samples in different combinations of macronutrient concentrations, different volumes of three macronutrient stock solutions were added together and diluted with demineralised water (MiliQ^®^ water purification system), resulting in the reported concentration ranges in Table 1.

For the carbohydrate stock solution, we used 100%*w*/*w* α-lactose monohydrate (L3625, Sigma Aldrich, Saint Louis, MO, USA). Oligosaccharides were not included because their relative contribution to the carbohydrate concentration is only 16%. With this lactose-based carbohydrate stock solution, we created different serum simulating samples with a final concentration ranging from 4.0 to 8.8 g/dL lactose in five steps of 1.2 g/dL to represent the natural range of carbohydrate concentrations in human milk serum.

For the whey protein stock solution, we used 35%*w*/*w* human lactoferrin (L1294, Sigma Aldrich) and 65%*w*/*w* bovine α-lactalbumin (L5385, Sigma Aldrich) because these two proteins make up the majority of the total whey proteins. Bovine α-lactalbumin was selected due to the unavailability of human α-lactalbumin. Since amino acid composition and bioactivity are similar between these two proteins [27], bovine α-lactalbumin is expected to have the same effect on the refractive index of milk serum as its human equivalent. The whey protein stock solution was used for the serum simulating samples with final whey protein concentrations ranging from 0.45 g/dL to 1.35 g/dL in four steps of 0.3 g/dL.

For the mineral stock solution, we used 25%*w*/*w* calcium chloride, 23%*w*/*w* sodium citrate dihydrate, and 52%*w*/*w* potassium hydroxide because these dissolved minerals make up more than 80% of all the minerals in human milk as can be seen from Table 1. This mineral stock solution completed the serum simulating samples with a final mineral concentration ranging from 0.090 to 0.210 g/dL in four steps of 0.040 g/dL.

**Table 1:** Composition of mature human milk serum (>30 days postpartum), based on the literature [8,9,28,29,30,31,32,33,34,35,36,37] and composition of the serum simulating samples (i.e., weight percent contribution of included constituents and simulated concentration range per macronutrient).

	Mature Human Milk Serum			Serum Simulating Samples	
↓ Macronutrient Constituent	Mean Concentration [g/dL]	Lowest Value [g/dL]	Highest Value [g/dL]	Included Constituents	Concentration Range [g/dL]
**Carbohydrates [30,33,36]**	**7.5**	**4.0**	**8.5**		**4.0–8.8**
Lactose [8,9,32]	6.3	2.4	8.5	100%*w*/*w* α-lactose	
Oligosaccharides [31,32]	1.2	1.0	1.5		
Glucose [30]	0.016	0.005	0.029		
**Whey proteins [30,32,37]**	**0.95**	**0.5**	**1.6**		**0.45–1.35**
α-lactalbumin [28,32]	0.30	0.26	0.30	65%*w*/*w*	
Lactoferrin [28,32]	0.18	0.15	0.20	35%*w*/*w*	
Immunoglobulin [28,32]	0.13	0.09	0.16		
**Minerals [29,32,34,35,36]**	**0.170**	**0.107**	**0.247**		**0.090–0.210**
Potassium [29,32,34]	0.058	0.030	0.091	52%*w*/*w* potassium hydroxide	
Chloride [29,32,35]	0.043	0.040	0.051	25%*w*/*w* calcium chloride	
Calcium [32,34,35]	0.027	0.014	0.043		
Sodium [29,35]	0.018	0.011	0.030	23%*w*/*w* sodium citrate dihydrate	
Phosphorus [29,32,35,36]	0.015	0.010	0.017		
Magnesium [32]	0.003	0.002	0.005		

### 2.3. Human Milk Samples

Nineteen breastfeeding volunteers aged between 27 and 39 years donated mature milk for this study. Ethical approval was obtained by the Natural Sciences and Engineering Sciences Ethics Committee of the University of Twente (reference-number 2021.118 and 230253, Enschede, The Netherlands) and all participants gave written consent prior to milk donation. Only healthy donors living in the Netherlands with a lactation period between 30 days and 9 months were included. More information about the donors and the lactation characteristics (i.e., time of milk expression, extracted milk volume, and time since previous feed) is presented in Table 2.

All volunteers followed a step-wise protocol for milk extraction and donated 7 mL of fore-, bulk-, and hindmilk from one breast, using their own breast pump. These three samples are defined as follows: (1) foremilk was defined as the first 7 to 15 mL of expressed breastmilk, (2) hindmilk was defined as the last 7 to 15 mL of expressed milk, and (3) bulk milk was defined as the milk expressed in between. Donor 10 and 18 deviated from the protocol by expressing a different volume of hindmilk, respectively, 1 mL and 35 mL.

### 2.4. Milk Preparation

After donation, the fore-, bulk-, and hindmilk samples were kept at room temperature and were prepared in the lab within four hours after donation for further analysis. An exception to this procedure were the milk samples from donors 2, 5, and 8. These milk samples were stored in the fridge (between 4 and 7 °C) for a maximum of 20 h before preparation in the laboratory. Each donated sample was split into different volumes: 3 mL milk for the mid-infrared human milk analyser, 0.25 mL milk for the refractometer measurement on whole (unskimmed) milk, and 0.62 mL milk for skimming and the refractometer measurement on skimmed milk. Both skimmed and whole (unskimmed) milk samples were evaluated because refractive index measurements with conventional prism based refractometers are most reliable for transparent fluid samples [38]. The transparency of skimmed milk is considerably higher compared to whole (unskimmed) milk, due to the removal of the majority of light scattering fat globules in the skimming procedure.

The milk was skimmed by centrifugation in three steps. First, 620 µL of milk was centrifuged for 5 min at 200× *g*. The fat layer was scooped off and 475 µL of the middle layer was transferred into a clean Eppendorf and centrifuged for 10 min at 1000× *g*. Again, the fat layer was skimmed and 350 µL of the middle layer was transferred into a clean Eppendorf and centrifuged for the last time for 20 min at 3000× *g*. 250 µL from this last middle layer was placed in a clean Eppendorf and was classified as skimmed milk. All skimming steps were performed at room temperature. Both the skimmed and whole (unskimmed) milk samples were frozen at −20 °C for a maximum of five months. Before the measurements, the samples were thawed at room temperature.

### 2.5. Refractometer

The refractive index of all samples was measured with a refractometer (Refracto 30GS, Mettler Toledo, Columbus, OH, USA) with an optical wavelength of 589.3 nm. A sample volume of 60 µL was pipetted onto the measuring cell of the refractometer. Subsequently the refractive index was measured three times per sample volume on the measuring cell. For the serum simulating samples, the pipetting procedure on the measuring cell was repeated three times, resulting in 9 measurements in total. For the whole and skimmed donated milk samples, the pipetting procedure was repeated four times, resulting in 12 measurements in total. The standard deviation of these 9 or 12 measurements per sample was defined as the error margin. In between successive measurements, the measurement cell was cleaned with a cleaning solution for human milk (Miris cleaner, Miris, Uppsala, Sweden), rinsed with water, and dried with cleaning tissue.

All measurements were performed between 20.1 and 23.9 °C. All values for the refractive index were corrected to a temperature of 22.0 °C using the relation between the refractive index at a wavelength of 589 nm and the temperature of water, as given by Equation (1) for temperatures in the range of 15–30 °C [39,40]. The temperature correction on the measured refractive index to 22.0 °C is calculated using Equation (2) with *T* the temperature of the measured sample in °C and *n* the refractive index of the sample at a specific temperature.

(1) $$n_{\;water}^{T}=1.334012-1.426729\times 10^{-5}\times T-1.831785\times 10^{-6}\times T^{2}\;$$

(2) $$n_{measured}^{22\;{}^{\circ}C}=n_{measured}^{T}+\left(n_{water}^{22\;{}^{\circ}C}-n_{water}^{T}\right)$$

### 2.6. Milk Analyser

The macronutrient composition of human milk was measured using a mid-infrared human milk analyser (Miris HMA, Uppsala, Sweden). This human milk analyser measures the fat, carbohydrate, true protein (i.e., an approximation of the concentration of whey proteins and caseins together) and total solids concentration of human milk in g/dL. The solids-not-fat concentration (i.e., the total contribution of carbohydrates, proteins, and minerals present in milk) is calculated by subtracting the fat concentration from the total solids concentration.

Prior to analysis, the human milk analyser was calibrated with a validation sample (Miris Check solution) and the calibration kit provided by the manufacturer, according to the operation instructions. For the macronutrient analysis, 3 mL of thawed milk was warmed to 40 °C in 30 min, homogenised for 5–6 s using the ultrasonic processor (Miris HMA, Sweden), and measured using the human milk analyser. The hindmilk samples were diluted two times with demineralized water up to a total volume of 4 mL prior to homogenization to assure that the higher fat concentration of hindmilk was within the measurement range of the milk analyser. The measured macronutrient concentrations for hindmilk were corrected for this dilution step. The human milk analyser was cleaned with a cleansing fluid (Miris cleaner) after the measurement of ten successive milk samples.

### 2.7. Statistical Analysis

Statistical analyses were performed in MATLAB (R2020a). The Spearman correlation was calculated for the relation between the refractive index and the macronutrient concentrations, as well as the relation between the refractive index and the donor specific information (lactation characteristics). This information includes the age of the donor, the expression time during the day, the expressed milk volume, the time since previous feed, the sex of the infant, and the lactation period. *p*-values below 0.05 were considered statistically significant and *p*-values between 0.05 and 0.1 were considered to indicate a weak correlation. Exact values were reported.

### 2.8. Analysis of Serum Simulating Samples

Water with dissolved carbohydrates, whey proteins, and minerals has an increased refractive index with respect to pure water. The total increase in refractive index depends on the concentration and the molecular species. We analysed the relation between the refractive index and the concentration of each constituent in the serum simulating samples by fitting a linear model to the data. For the carbohydrates, this fit was performed on the refractive index against the carbohydrate concentration for constant mineral and whey protein concentration:

(3) $$n_{sample}=a\times \left[carbohydrate\right]+b+n_{water\;}$$

Here, *n* is the refractive index of the sample at λ = 589.3 nm, *a* is the slope of the linear fit, which is related to the refractive index increase due to the carbohydrate concentration, [*carbohydrate*], of the sample. The parameter *b* is the refractive index increase with respect to pure water, caused by the presence of whey proteins and minerals in the serum simulating sample, and *n_water_* is the refractive index of pure water (1.33281 for λ = 589.3 nm at 22 °C).

To analyse the relation between the refractive index increase caused by the whey protein concentration in the sample, we also performed a linear fit on the parameters *b* from Equation (3) against the whey protein concentration:

(4) $$b=c\times \left[protein\right]+d$$

Here, *c* is the slope of the linear fit with units per g/dL. *c* is related to the refractive index increase with respect to pure water, due to the whey protein concentration, [*protein*], of the sample. The parameter *d* is the offset, which is related to the mineral concentration of the sample.

## 3. Results

### 3.1. Serum Simulating Samples

**Figure 1:**
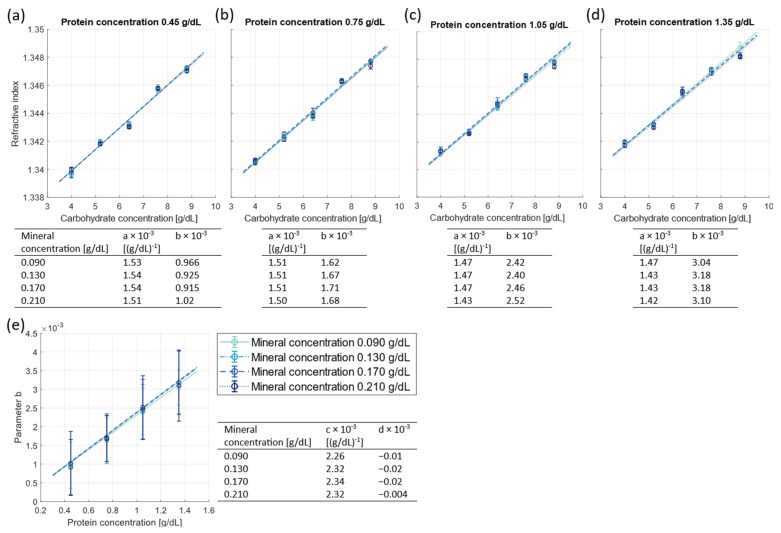
(**a**–**d**) the measured refractive index (o) of the serum simulating samples as a function of the carbohydrate concentration, for different mineral concentrations (0.090, 0.130, 0.170, and 0.210 g/dL). Between the four different subplots, the protein concentration is varied (0.45, 0.75, 1.05, and 1.35 g/dL). The lines are linear fits on the refractive index against the carbohydrate concentration per constant mineral and protein concentration, with the slope *a* and offset *b* displayed in the tables below each graph. The error bars represent the standard deviation of the measurements and may fall behind data points. (**e**) The parameter *b* from the tables below graphs a-d as a function of the protein concentration. The lines are linear fits on the parameter *b* against the protein concentration with slope *c* and offset *d*, as displayed in the table on the right. The error bars represent the 95% confidence interval of the linear fit on the refractive index against carbohydrate concentration.

Figure 1a–d present the refractive index of the serum simulating samples as a function of carbohydrate concentration, for mineral concentrations ranging between 0.090 and 0.210 g/dL. Between the four different subplots, the whey protein concentration is varied from 0.45 to 1.35 g/dL. For each mineral and whey protein concentration, a linear fit was performed on the refractive index against carbohydrate concentration, according to Equation (3). The fitted parameters *a* and *b* are listed in the tables below each plot. The fit parameter *a* (slope) presents a measure for the dependency of the refractive index on the carbohydrate concentration, where the average parameter *a* is 1.48 × 10^−3^ ± 0.04 × 10^−3^ per g/dL carbohydrate. The fit parameter *b* (offset) presents a measure for the increase in refractive index with respect to pure water due to the presence of whey proteins and minerals in the sample.

Figure 1e presents this fit parameter *b* from Equation (3) as a function of the whey protein concentration per mineral concentration. This relation provides information on the dependency of the refractive index on the protein concentration. Using Equation (4), a linear fit was performed on the parameter *b* against the whey protein concentration. This resulted in the fit parameters *c* (slope) and *d* (offset). The average slope parameter *c* is 2.31 × 10^−3^ ± 0.05 × 10^−3^ per g/dL and the offset *d* is negligible.

In general, Figure 1 demonstrates that the refractive index of human milk serum simulating samples is influenced by commonly found variations in carbohydrate and whey protein concentrations. This is reflected in the fitted values for *a* and *c*: the increase in refractive index for 1 g/dL carbohydrate and 1 g/dL whey protein is 1.48 × 10^−3^ ± 0.04 × 10^−3^ and 2.31 × 10^−3^ ± 0.05 × 10^−3^, respectively. On the other hand, the refractive index is not influenced substantially by commonly found variations in mineral concentrations, since the parameters *b* (offset) and *d* (offset) do not consistently increase with increasing mineral concentration.

### 3.2. Human Milk Samples

**Figure 2:**
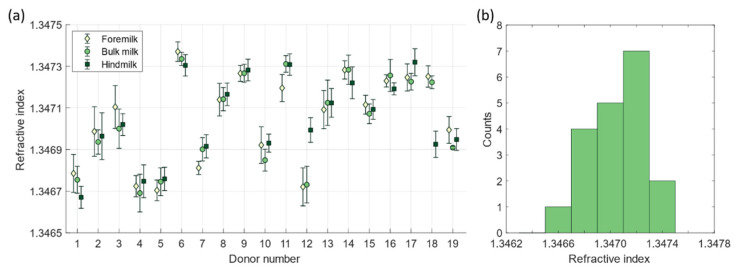
The refractive index of the donated human milk samples within (**a**) the measured refractive index per sample and (**b**) a histogram of the refractive index of all skimmed mature bulk milk samples.

Figure 2a presents the measured refractive index of all human milk serum samples that were obtained after skimming the donated milk samples. What stands out is that the refractive index of fore-, bulk, and hindmilk per donor is approximately the same, except for donor 12 and 18, where the refractive index of hindmilk differs from fore- and bulk milk. Furthermore, the refractive index differs between donors and ranges from 1.3466 to 1.3474. The range in the refractive indices of all skimmed bulk milk samples is presented in the histogram of Figure 2b. The histogram approximates a normal distribution, with an average refractive index of 1.3470.

**Figure 3:**
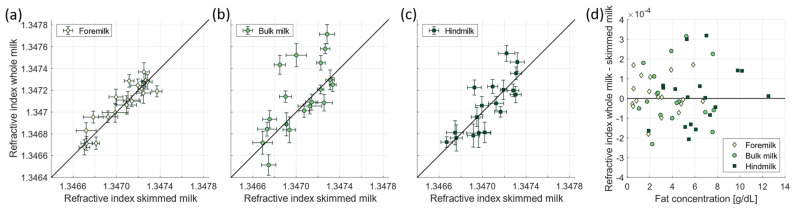
The measured refractive index of whole milk samples as a function of the measured refractive index of skimmed milk samples for (**a**) foremilk, (**b**) bulk milk, and (**c**) hindmilk. (**d**) The difference in measured refractive index between the whole milk and skimmed milk samples as a function of fat concentration of the whole milk sample.

Figure 3a–c present the measured refractive index of the whole milk samples against the measured refractive index of the skimmed milk samples per donor to visualise the effect of the presence of milk fat globules on the accuracy of the refractive index measurements. In case of foremilk (Figure 3a), the measurements of whole milk give comparable results to the measurements of skimmed milk (Spearman correlation coefficient R = 0.89, *p* = 4 × 10^−7^). For bulk milk and hindmilk (Figure 3b,c), the correlation is still significant (R = 0.69, *p* = 0.0015, and R = 0.78, *p* = 0.00014, respectively), but more than half of the whole milk measurements deviate more than a standard deviation (error bar) from the skimmed milk measurements. Since the main difference in macronutrient composition between fore-, bulk, and hindmilk is the fat concentration [41] and thereby the concentration of light scattering particles. Figure 3d presents the relation between the difference in measured refractive index of the whole and skimmed milk samples against the fat concentration of the whole milk samples, as measured with the human milk analyser. Counter to our expectation, Figure 3d does not show a significant relation between the difference in measured refractive index and the fat concentration (R = −0.06, *p* = 0.66).

**Figure 4:**
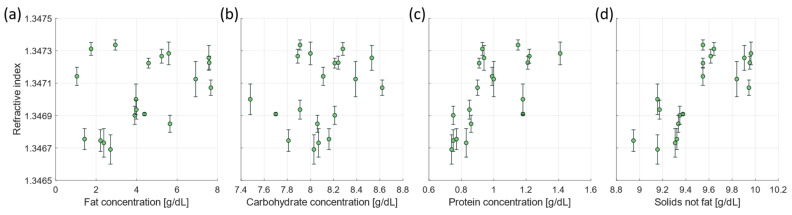
The refractive index of skimmed bulk milk samples as a function of (**a**) fat concentration, (**b**) carbohydrate concentration, (**c**) protein concentration, (**d**) and the solids-not-fat concentration.

Figure 4 presents the relation between the measured refractive index of the skimmed bulk milk samples and the macronutrient composition, as measured by the human milk analyser. The measured refractive index correlates significantly with the protein concentration (Spearman correlation coefficient R = 0.75, *p* = 0.00025) and the solids-not-fat concentration (R = 0.76, *p* = 0.00014). No significant correlation was observed for the relation between the refractive index and the carbohydrate concentration (R = 0.21, *p* = 0.39) nor the fat concentration (R = 0.30, *p* = 0.21).

**Figure 5:**
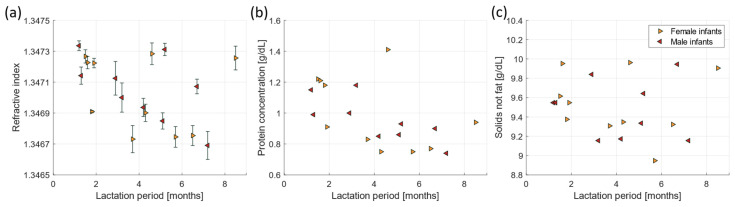
(**a**) Refractive index, (**b**) protein concentration, and (**c**) the solids-not-fat as a function of lactation period. The legend in figure c applies to a and b as well.

In addition to the macronutrient composition, we investigated the correlation between the measured refractive index of skimmed bulk milk and donor specific information (lactation characteristics), including the age of the donor, the expression time during the day, the expressed milk volume, the time since previous feed, the sex of the infant, and the lactation period. Only the lactation period shows a weak correlation with the refractive index (R = −0.39, *p* = 0.097, Figure 5a), where the refractive index decreases over the course of lactation. This finding can be related to a significant decrease in protein concentration over course of lactation (R = −0.59, *p* = 0.0075), as shown in Figure 5b. This relation is not influenced by the sex of the infant. One milk sample at 4.5 months of lactation has a relatively high protein concentration (1.41 g/dL) compared to the other samples. The donor of this sample indicated that her infant had a viral infection at the time of milk donation. The solids-not-fat concentration did not show a correlation with the lactation period (R = −0.15, *p* = 0.55), as shown in Figure 5c.

## 4. Discussion

The main goal of this study was to identify the relation between the macronutrient composition of human milk serum and its refractive index. We measured the refractive index of serum simulating samples with known concentrations of carbohydrates, whey proteins, and minerals to analyse the influence of each dissolved constituent on the refractive index of milk serum. We also measured the refractive index of whole and skimmed human milk samples and evaluated the relation between the measured refractive index and the macronutrient composition. In general, the refractive index is related to the density of a material, because closer packed molecules have a larger effect on the propagation speed of light. The presence of particles such as milk fat globules and casein micelles does not affect the refractive index of the milk serum since they are in the dispersed phase. Instead, milk fat globules and casein micelles cause light scattering, which makes a sample opaque.

### 4.1. Serum Simulating Samples

Our serum simulating samples were designed to represent biological variations in carbohydrate, whey protein, and mineral concentrations. We found that the refractive index of these serum simulating samples was only influenced by variations in carbohydrate and whey protein concentrations and that the mineral concentration did not substantially influence the refractive index. Variations in the whey protein concentration had the largest influence on the refractive index. Therefore, the refractive index of milk can potentially be used to predict changes in the chemical composition of milk serum, although a combination with other methods will remain necessary to predict the individual concentrations of dissolved proteins and carbohydrates.

The serum simulating samples in this study were designed such that they represent the presence of the most abundant dissolved constituents in human milk serum (Table 1). A limitation of this study is that the serum simulating samples had a whey protein concentration ranging from 0.45 to 1.35 g/dL, while extreme protein concentrations of 1.6 g/dL have been reported in the literature [42]. Creating serum simulating samples with higher whey protein concentrations was hindered by the solubility of α-lactalbumin and lactoferrin. Future studies could include other proteins such as serum albumin or immunoglobulins to reach higher total protein concentrations in the serum simulating samples. Since human milk samples with a whey protein concentration higher than 1.30 g/dL are rare [37], we argue that the range of protein concentrations of the serum simulating samples is an adequate approximation for human milk. This is also reflected by the protein concentrations in the donated milk samples, in which only one sample exceeds a total protein concentration of 1.35 g/dL.

Another limitation is that not all serum constituents (Table 1) were represented in the serum simulating samples, such as IgA, free caseins, or oligosaccharides. Therefore, the influence of these less abundant constituents on the refractive index of human milk serum was not evaluated in this study. Nevertheless, the range in measured refractive indices of the serum simulating samples (1.3397–1.3487) well encompassed the total range in measured refractive indices of the donated human milk samples (1.3466–1.3474). The larger range in refractive indices of the serum simulating samples can be attributed to the larger range in simulated carbohydrate (4.0–8.8 g/dL) and whey protein concentrations (0.45–1.35 g/dL), compared to the measured carbohydrate (7.48–8.62 g/dL) and total protein concentrations (0.74–1.41 g/dL) in the donated milk samples.

### 4.2. Human Milk Samples

The measured refractive index of human milk serum in the donated samples was on average 1.3474 (1.3466–1.3474). The refractive index of fore-, bulk, and hindmilk per donor is approximately the same, which can be explained by the fact that the carbohydrate and protein concentration do not vary significantly during a single breastfeed [41]. However, the hindmilk samples of donor 12 and 18 differed in refractive index from their foremilk and bulk milk samples. This result could not be related to any of the recorded lactation characteristics (milk expression time, lactation period, total expressed milk volume, etc.). The refractive index of human milk serum measured in this study is slightly lower compared to the refractive index of whole human milk samples measured using Abbe’s refractometer, as reported by Sunarić et al. [23] who measured values between 1.3482 and 1.3510. Furthermore, the measured refractive index of human milk serum in this study is comparable to the refractive index values of milk serum from other mammalian species, such as bovine milk serum of 1.3451 and buffalo milk serum of 1.3472 [43], but the range of measured refractive indices is smaller compared to the values reported for bovine, buffalo [43] and sheep milk [44].

The milk samples donated by donors 2, 5, and 8 were stored in the fridge for a maximum of 20 h before preparation in the laboratory, while the other donated milk samples were prepared within four hours after expression. These refrigerated samples were not excluded from the dataset, as the refrigeration of milk at 4 °C for 72 h has minimal impact on the total protein, carbohydrate, and mineral concentrations in milk serum [45]. Our results also demonstrate that the measured refractive indices of these refrigerated milk samples fall within the range of measured refractive indices from the non-refrigerated milk samples.

The highly abundant light scattering by milk fat globules [11] causes refractive index measurements with conventional prism-based refractometers to be more difficult to perform [38]. Whereas skimming removes the majority of milk fat globules, refractive index measurements on whole milk are less destructive to the sample and less labour intensive. Therefore, we compared the measured refractive index of skimmed to whole milk samples. This comparison revealed that the measurements on whole, and skimmed milk samples are comparable. However, this agreement is better for foremilk, compared to bulk and hindmilk. The main difference between fore-, bulk, and hindmilk is the fat concentration, with the lowest fat concentration in foremilk and the highest fat concentration in hindmilk. Unexpectedly, we did not observe a relation between the fat concentration and the difference in measured refractive index between whole and skimmed milk (Figure 3d). We speculate that this lack of correlation can potentially be related to differences in milk fat globule size distributions between these samples [46,47] as the light scattering behaviour of whole milk is known to be influenced by both the size and particle concentration of milk fat globules [48]. To better understand this observation, other optical techniques to measure the refractive index that are less sensitive to sample turbidity could be employed, such as a U-bent optical fibre probe [49] or optical waveguide based sensors [50].

The measured refractive index correlated significantly with the total protein concentration (R = 0.75) and the solids-not-fat concentration (R = 0.76) of the donated milk. This is consistent with the work of Rangappa et al. [43] who also observed a relation between the refractive index and the solids-not-fat concentration for buffalo and cow milk. As the solids-not-fat concentration of bovine and buffalo milk is diet related [51,52], the measurement of solids-not-fat in human milk might provide a more in-depth understanding in its relation to maternal diet and the milk synthesis and secretion process by the mammary gland. For this study, we expect that the dependency of the refractive index on the solids-non-fat concentration is mostly influenced by the protein concentration due to the similar behaviour (Figure 4c,d) and similar correlation coefficients for both parameters with refractive index. A limitation of this study is that the human milk analyser measures the total protein concentration, which is the sum of caseins and whey proteins, whereas the refractive index only depends on whey proteins. Consequently, the presented relation between the refractive index and the protein concentration (Figure 4c) is affected by the non-dissolved casein concentration in milk. For mature human milk from donors with a lactation period between 1 and 9 months, the casein concentration ranges between 18 and 45%, with an average of 25.5% with respect to the total protein concentration [42]. Therefore, the correlation between the refractive index and the protein concentration is most likely even stronger in case caseins are not included in the comparison. For future studies, it is recommended to measure both the whey protein and casein concentration individually. This can be achieved by separation of the caseins from the milk serum, for example, through acidification and centrifugation [42].

Surprisingly, the carbohydrate concentration did not show a significant correlation with the refractive index in the donated milk samples, although this was expected based on the carbohydrate concentration dependency of the refractive index in the serum simulating samples. This can be explained by the smaller range in carbohydrate concentrations in the donated milk samples (7.5–8.6 g/dL) compared to the serum simulating samples (4.0–8.8 g/dL). It is expected that a larger dataset that includes milk samples with a wider range of carbohydrate concentrations will reveal the correlation between the refractive index and the carbohydrate concentration in human milk. In addition, the human milk analyser has a limited accuracy for the carbohydrate concentration determination. This accuracy is 13% as reported by the manufacturer [53] but validation studies in the literature suggest that this accuracy can be lower in the practice of human milk analysis [6]. It is recommended for future studies to measure the carbohydrate concentration of human milk serum using other more accurate methods, such as high pressure liquid chromatography (HPLC) [7,8].

For the donated milk samples, we observed a significant decrease in the refractive index as a function of lactation period. This finding can be explained by a significant decrease in protein concentration as a function of lactation period. This is consistent with previous studies by Liao et al. [42], Zhang et al. [37], and Saarela et al. [41], who all observed a significant decrease in protein concentration over the course of lactation.

## 5. Conclusions

In this study, we reported on the relation between human milk serum composition and its refractive index. In serum simulating samples, the refractive index depended more on the whey protein concentration (2.31 × 10^−3^ ± 0.05 per g/dL proteins) than the carbohydrate concentration (1.48 × 10^−3^ ± 0.04 per g/dL carbohydrates). Furthermore, biological variations in the mineral concentration of human milk serum did not substantially influence the refractive index. These serum simulating samples well predicted the measured refractive index of the donated human milk samples for this study, which ranged between 1.3466 and 1.3474, with an average of 1.3470. Per donor, no significant differences were observed in the refractive index between fore-, bulk, and hindmilk. A significant, but moderate correlation was observed between the refractive index and the protein concentration of the donated milk samples, as well as the solids-not-fat concentration. In conclusion, this study contributes to adequate data interpretation for various optical methods, which use light scattering to analyse milk composition.

## Figures and Tables

**Table 2:** Milk donor specific information.

Donor Number	Age Donor	Lactation Period (Months)	Time of Milk Expression	Total Extracted Milk Volume (mL)	Time Since Previous Feed (Hours)	Sex Infant
1	34	6.5	11:00	100	11	F
2	31	4.2	21:30	85	3.5	M
3	37	3.2	15:15	80	1	M
4	35	7.2	12:15	150	5	M
5	28	5.7	16:15	105	7	F
6	33	1.2	09:30	85	3	M
7	34	4.3	11:45	45	2.5	F
8	30	1.3	13:30	90	3	M
9	37	1.5	09:30	105	4	F
10	27	5.1	12:15	130	4	M
11	33	5.2	10:00	155	4	M
12	39	3.7	09:30	35	2.5	F
13	30	2.9	10:00	80	2.25	M
14	29	4.6	14:15	27	2.25	F
15	32	6.7	10:40	75	3.5	M
16	31	8.5	15:45	70	7	F
17	28	1.6	10:20	85	3.5	F
18	30	1.9	11:00	80	4.2	F
19	31	1.8	10:30	45	2	F

## Data Availability

The original contributions presented in the study are included in the article, further inquiries can be directed to the corresponding author.
